# Effects of different treatment methods on clinical efficacy and fertility outcomes of patients with adenomyosis

**DOI:** 10.1186/s13048-023-01320-0

**Published:** 2024-01-12

**Authors:** Zhaoping Chu, Ligang Jia, Jun Dai, Qi Wu, Fei Tian, Suning Bai

**Affiliations:** 1 Department of Gynaecology, He Bei General Hospital, 348 Heping West Road, Shijiazhuang, 050051 Hebei China; 2 Department of Immunology and Pathobiology, Hebei University of Chinese Medicine, Shijiazhuang, 050200 Hebei China

**Keywords:** Adenomyosis, Ultrasonic guidance, Percutaneous radiofrequency ablation, Leuprorelin acetate, Laparoscopic surgery

## Abstract

**Objective:**

This trial was to investigate the effect of different treatment methods on the clinical efficacy and fertility outcome of patients with adenomyosis.

**Methods:**

In total, 140 patients with adenomyosis were evenly and randomly allocated into group A (laparoscopic surgery), group B (laparoscopic surgery combined with gonadotropin-releasing hormone analogs [GnRH-a]), group C (ultrasound-guided percutaneous radiofrequency ablation), and group D (ultrasound-guided percutaneous radiofrequency ablation combined with GnRH-a). On the 3rd day after surgery, patients in group B and group D were subcutaneously injected with GnRH-a (Leuprorelin Acetate SR for Injection) at 3.75 mg/time, once every 4 weeks, for a total of 3 months. The therapeutic effects of the 4 groups were compared, including menstrual volume, dysmenorrhea score, uterine volume, clinical efficacy, luteinizing hormone (LH), estradiol (E2), and follicle-stimulating hormone (FSH) levels, CA125 levels, recurrence, pregnancy status, and pregnancy outcomes.

**Results:**

After treatment, the menstrual volume of 4 groups was lowered, dysmenorrhea, Visual Analog Scale (VAS) score, LH, FSH, E2, and CA125 levels were reduced, and uterine volume was decreased. The menstrual volume, VAS score, levels of LH, FSH, E2, and CA125, and uterine volume were reduced in groups B, C, and D compared with group A, and the decrease was more significant in group D. The total effective rate of group D was 100.00%, which was higher than that of group A (71.43%), group B (80.00%), and group C (82.86%). After one year of drug withdrawal, the recurrence of hypermenorrhea, dysmenorrhea, uterine enlargement, and excessive CA125 in group D was significantly lower than that in groups A, B and C, and the recurrence in groups B and C was significantly lower than that in group A (*P* < 0.05). Compared with groups A, B, and C, group D had a higher pregnancy rate, natural pregnancy rate, and lower in vitro fertilization-embryo transfer rate (*P* < 0.05), but showed no significant difference in pregnancy outcomes.

**Conclusion:**

Ultrasound-guided percutaneous radiofrequency ablation combined with Leuprorelin Acetate is effective in the treatment of adenomyosis, which can effectively relieve clinical symptoms, protect postoperative ovarian function, reduce recurrence rate, alleviate pain, and improve quality of life.

## Introduction

Adenomyosis is a common gynecological disease that clinically causes abnormal uterine bleeding, pelvic pain, and infertility [1]. It is characterized by the abnormal appearance of endometrial epithelial cells and interstitial fibroblasts, causing the proliferation and hypertrophy of surrounding smooth muscle cells [2]. A growing number of papers have shown that not only fertility but also pregnancy outcomes are affected by adenomyosis. Given late pregnancy outcomes, patients with adenomyosis are at an increased risk of preterm delivery and premature rupture of membranes [3, 4]. Therefore, it is a clinical requirement to improve pregnancy outcomes and treatment efficacy in women with adenomyosis.

The standard treatment for adenomyosis is hysterectomy, but there is currently no drug treatment that can treat the symptoms of adenomyosis while still enabling patients to get pregnant [5]. Since hysterectomy is not an option for women wishing to preserve fertility, interventions to preserve the uterus, especially minimally invasive treatments, have been advocated [6, 7]. To preserve fertility and relieve symptoms, medication is usually the first choice, while for refractory adenomyosis, surgical resection may be performed, but pregnancy rates vary widely after conservative surgical treatment [8]. Ultrasound-guided radiofrequency ablation (RFA) has been appraised due to its safety and effectiveness in the treatment of adenomyosis [9]. Abnormal sex steroid signaling is known to play a key role in adenomyosis, which is why various anti-estrogen drugs have been used to treat adenomyosis symptoms [10]. Among them, gonadotropin-releasing hormone analogs (GnRH-a) are making rapid progress [11, 12]. Medications that act as agonists and antagonists of GnRH are effective in treating hormone-dependent disorders due to their regulation of the hypothalamic-pituitary-gonadal axis [13]. It has been reported that GnRH-a can temporarily improve symptoms in patients with adenomyosis [14] and can improve pregnancy outcomes in those with diffuse adenomyosis [15]. Meanwhile, reports have considered and confirmed the feasibility and application of GnRH-a as a combined treatment method to reduce adenomyotic lesions and alleviate symptoms [16, 17].

Based on previous studies, this trial was conducted to explore the effect of different treatment methods on clinical efficacy and pregnancy outcomes of patients with adenomyosis to find a more effective way to improve adenomyosis.

## Materials and methods

### Ethics statement

This study was approved by He Bei General Hospital ethics committee, and patients or their families gave informed consent and signed informed consent.

### Subjects

A total of 140 patients with adenomyosis admitted to He Bei General Hospital from January 2020 to December 2021 were randomized into group A (laparoscopic surgery), group B (laparoscopic surgery combined with gonadotropin-releasing hormone analogs [GnRH-a]), group C (ultrasound-guided percutaneous radiofrequency ablation), and group D (ultrasound-guided percutaneous radiofrequency ablation combined with GnRH-a), with 25 cases in each group.

Inclusion criteria: (1) Patients were diagnosed with adenomyosis after an ultrasound or magnetic resonance imaging examination, which was consistent with the relevant diagnostic criteria in Obstetrics and Gynecology: ① Clinical manifestations were dysmenorrhea and hypermenorrhea, which had been aggravated; ② Gynecological examination indicated uterine enlargement; ③ Vaginal color ultrasonography indicated that the uterus was enlarged, there were abundant blood flow signals in the uterine wall, manifested as diffuse congestion, and color signals existed in the liquid anechoic area; (2) Patients had fertility requirements and did not use any contraceptive and sex hormone drugs within 3 months before surgery; (3) Patients met the criteria for laparoscopic focal resection.

Exclusion criteria: (1) Patients with cervical and endometrial lesions; (2) Patients with mental illness; (3) Patients with metabolic, immune, and endocrine-related diseases; (4) Patients with significantly decreased ovarian function; (5) Patients with hypoestrogen and hypocalcemia; (6) Patients with allergies; (7) Patients with ovarian cysts and uterine fibroids.

### Treatment methods

Patients in Group A underwent laparoscopic surgery, which was performed by 2 professional physicians. Laparoscopy was implanted after Trocar routine puncture, and preoperative ultrasound and MRI imaging data were combined with intraoperative exploration. The spindle incision of the diseased tissue was excised as thoroughly as possible in a location where the lesion was evident, and the lesion was removed without penetrating the uterine wall. Muscle layer and seromuscular layer were sutured to stop bleeding and repair the uterus. No dead space was found during surgery.

Based on group A, patients in group B were given subcutaneous injection of Leuprorelin Acetate (Shanghai Livzon Pharmaceutical Co., Ltd., H20093852) at 3.75 mg each time, once every 4 weeks, for 3 months after pathological diagnosis on the 3rd day after surgery.

Patients in Group C underwent ultrasound-guided percutaneous RFA. Lesion size, location, boundary, and blood supply were evaluated by contrast-enhanced ultrasound in patients posed in a supine position. In the treatment area, the skin was prepared, an indwelling catheter was placed, and venous access was opened. In the case of the lagedes uterus, it could be adjusted and fixed by a transvaginal probe. Routine disinfection was performed, and the intestines and bladder were avoided under ultrasound guidance (appropriate pressure of the probe could squeeze the intestine out). The microwave needle was inserted into the target lesion and ablation began. If the treatment area occurred vaporization, the needle was withdrawn to the surface of the lesion and then moved to enter the lesion again by wigging the needle handle, and a multi-angle puncture was performed until the lesion was completely covered by the treatment area. If the maximum diameter of the lesion was ≥ 5 cm, double-needle ablation could be used. When the treatment area was close to the bowel, artificial ascites could be established to isolate the surrounding bowel and bladder to avoid thermal damage to the surrounding tissue. An intraoperative electrocardiogram was performed to monitor the patient’s vital signs.

On the basis of group C, patients in group D were given subcutaneous injections of Leuprorelin Acetate (Shanghai Livzon Pharmaceutical Co., Ltd., H20093852) at 3.75 mg each time, once every 4 weeks, for 3 months after pathological diagnosis on the 3rd day after surgery.

### Observation indices

1. Dysmenorrhea was evaluated by the Visual Analog Scale (VAS). 0 indicates no pain and 10 indicates the most intense pain.
2. Uterine volume was measured by vaginal ultrasound before and 3 months after treatment (uterine volume = 0.52 × long diameter × anteroposterior diameter × transverse diameter).
3. Menstrual volume was assessed by Policy-Based Access Control (PBAC).
4. Clinical effect was determined. Obviously effective: uterine volume reduced, the focal echo was significantly enhanced, and the clinical symptoms such as dysmenorrhea, menstrual disorders, and painful sexual intercourse were significantly improved; Effective: uterine volume decreased, the echo of the lesion area was moderately enhanced, and the clinical symptoms were partially improved. Ineffective: Uterine volume did not shrink or increase after treatment, and clinical symptoms did not improve significantly. Total effective rate = (cases of obviously effective + effective)/total cases × 100%.
5. Hormone levels: Before surgery and 3 months after surgery, peripheral venous blood was collected from patients on the 3rd day of menstruation to determine luteinizing hormone (LH), follicle-stimulating hormone (FSH), and estrogen (E2).
6. Fasting venous blood (5 mL) was collected before surgery and 3 months after surgery, respectively, and serum CA125 was determined by radioimmunoassay.

Recurrence: At 12 months after drug withdrawal, the recurrence of dysmenorrhea, hypermenorrhea, excessive CA125, and uterine enlargement were compared.

7. Pregnancy status and pregnancy outcomes (miscarriage, premature birth, and full-term birth) were recorded.

### Statistical analysis

Data were processed by SPSS 20.0 statistical software. Measurement data were expressed as mean ± standard deviation and compared by t-test or one-way analysis of variance. Enumeration data were expressed as n or n (%) and compared by χ^2^ test. *P* < 0.05 was considered statistically significant.

## Results

### General data

The four groups did not differ significantly in terms of age, course of disease, pregnancy number, location of adenomyoma, endometrial thickness, combined chocolate cyst, abnormal menstruation, and dysmenorrhea (*P* > 0.05, Table 1).

**Table 1:** Comparison of general data

	Group A (n = 35)	Group B (n = 35)	Group C (n = 35)	Group D (n = 35)	*P*
Age (years)	32.74 ± 3.90	32.37 ± 3.65	32.54 ± 3.21	33.379 ± 3.75	0.68
Course of disease (years)	2.17 ± 0.79	2.11 ± 0.72	2.09 ± 0.74	2.06 ± 0.68	0.926
Pregnancy number (times)	2.09 ± 0.66	2.09 ± 0.82	2.14 ± 0.77	2.17 ± 0.75	0.952
Adenomyoma location (case)					0.947
Anterior wall	9	11	11	8	
Posterior wall	20	20	18	20	
Uterine fundus	6	4	6	7	
Endometrial thickness (mm)	8.62 ± 1.23	8.79 ± 1.43	8.37 ± 1.36	8.93 ± 1.32	0.337
Combined chocolate cyst	11	13	12	15	0.782
Abnormal menstruation	11	11	10	10	0.948
Dysmenorrhea	21	22	20	22	0.896

### Dysmenorrhea degree, menstrual volume, and uterine volume

Menstrual volume, dysmenorrhea degree, and uterine volume did not differ significantly between the four groups before treatment (*P* > 0.05). After treatment, menstrual volume, VAS score, and uterine volume in 4 groups were all lower than before treatment (*P* < 0.05). Menstrual volume, VAS score, and uterine volume of groups B, C and D were lower than those of group A, while those were lower in group D than those of groups B and C (*P* < 0.05, Table 2).

**Table 2:** Comparison of VAS scores, menstrual volume, and uterine volume

Groups	Time	VAS score	Menstrual volume (ml)	Uterine volume (cm^3^)
Group A (n = 35)	Before treatment	5.46 ± 1.29	296.76 ± 31.19	216.17 ± 10.80
	After treatment	2.89 ± 0.93*	104.72 ± 12.86*	155.91 ± 7.24*
Group B (n = 35)	Before treatment	5.66 ± 1.26	291.52 ± 30.66	218.32 ± 11.78
	After treatment	1.80 ± 0.68*#	85.43 ± 8.26*#	135.08 ± 6.23*#
Group C (n = 35)	Before treatment	5.69 ± 1.43	296.28 ± 30.84	217.13 ± 10.26
	After treatment	1.89 ± 0.63*#	86.34 ± 8.11*#	136.46 ± 6.20*#
Group D (n = 35)	Before treatment	5.43 ± 1.31	294.21 ± 31.05	218.12 ± 10.87
	After treatment	0.66 ± 0.48*#$&	52.55 ± 5.03*#$&	103.30 ± 5.21*#$&

Note: * *P* < 0.05 vs. before treatment; # *P* < 0.05 vs. group A; $ *P* < 0.05 vs. group B; & *P* < 0.05 vs. group C

### Curative effect

The total effective rate of group D was 100.00%, which was higher than that of group A (71.43%), group B (80.00%), and group C (82.86%) (*P* < 0.05, Table 3).

**Table 3:** Comparison of curative effects

Groups	Obviously effective	Effective	Ineffective	Total effective rate
Group A (n = 35)	12 (34.29%)	13 (37.14%)	10 (28.57%)	71.43
Group B (n = 35)	16 (45.71%)	12 (34.29%)	7 (20.00%)	80
Group C (n = 35)	17 (48.57%)	12 (34.29%)	6 (17.14%)	82.86
Group D (n = 35)	24 (68.57%)	11 (31.43%)	0	100.00*#$

Note: * *P* < 0.05 vs. group A; # *P* < 0.05 vs. group B; $ *P* < 0.05 vs. group C

### Hormone levels

Hormone levels prior to treatment did not differ statistically significantly among the 4 groups (*P* > 0.05), and they all decreased significantly after treatment (*P* < 0.01). After treatment, LH, FSH, and E2 in groups B, C and D were lower than those in group A, and they were lower in group D than those in groups B and C (*P* < 0.01, Table 4).

**Table 4:** Comparison of hormone levels

Groups	Time	Luteinizing hormone (IU/L)	Follicle-stimulating hormone (IU/L)	Estradiol (pmol/L)
Group A (n = 35)	Before treatment	18.91 ± 2.13	15.02 ± 2.21	288.17 ± 32.85
	After treatment	16.50 ± 1.85*	13.44 ± 1.55*	236.22 ± 22.38*
Group B (n = 35)	Before treatment	18.23 ± 2.18	14.92 ± 2.16	289.52 ± 32.14
	After treatment	14.96 ± 1.69*#	10.16 ± 1.27*#	206.93 ± 21.66*#
Group C (n = 35)	Before treatment	18.46 ± 2.17	15.09 ± 2.32	286.75 ± 33.55
	After treatment	14.28 ± 1.61*#	11.31 ± 1.31*#	209.40 ± 22.68*#
Group D (n = 35)	Before treatment	18.70 ± 2.45	15.41 ± 2.13	285.07 ± 30.21
	After treatment	11.40 ± 1.19*#$&	9.33 ± 1.07*#$&	183.37 ± 17.81*#$&

Note: * *P* < 0.05 vs. before treatment; # *P* < 0.05 vs. group A; $ *P* < 0.05 vs. group B; & *P* < 0.05 vs. group C

### CA125 levels

Before treatment, no statistically significant differences were found among the 4 groups in terms of CA125 levels (*P* > 0.05), and CA125 was notably decreased after treatment (*P* < 0.01). After treatment, CA125 level in groups B, C and D was markedly decreased compared with group A, and CA125 level in group D was distinctly decreased compared with groups B and C (*P* < 0.01, Table 5).

**Table 5:** Comparison of CA125 levels

Groups	CA125 (U/ml)	
	Before treatment	After treatment
Group A (n = 35)	52.42 ± 13.02	42.58 ± 12.13*
Group B (n = 35)	50.90 ± 13.07	35.50 ± 9.46*#
Group C (n = 35)	52.05 ± 13.24	33.91 ± 9.74*#
Group D (n = 35)	51.36 ± 13.48	21.52 ± 8.12*#$&

Note: * *P* < 0.05 vs. before treatment; # *P* < 0.05 vs. group A; $ *P* < 0.05 vs. group B; & *P* < 0.05 vs. group C

### Recurrence

After 12 months of drug withdrawal, the recurrence of hypermenorrhea, dysmenorrhea, uterine enlargement, and excessive CA125 in group D was significantly lower than that in groups A, B, and C (*P* < 0.01, Table 6).

**Table 6:** Comparison of recurrence

Groups	Excessive CA125	Uterine enlargement	Dysmenorrhea	Hypermenorrhea
Group A (n = 35)	15 (42.85%)	15 (42.85%)	16 (45.71%)	15 (42.85%)
Group B (n = 35)	11 (31.43%)	8 (22.86%)	10 (28.57%)	13 (37.14%)
Group C (n = 35)	12 (34.29%)	10 (28.57%)	9 (25.71%)	11 (31.43%)
Group D (n = 35)	3 (8.57%)*#$	1 (2.86%)*#$	2 (5.71%)*#$	3 (8.57%)*#$

Note: * *P* < 0.05 vs. group A; # *P* < 0.05 vs. group B; $ *P* < 0.05 vs. group C

### Pregnancy outcomes

The pregnancy rate of group D was significantly higher than that of groups A, B and C. No significant differences were found in pregnancy outcome, but group D posted a significantly higher rate of spontaneous pregnancy and the in vitro fertilization-embryo transfer rate was lower than groups A, B, and C (*P* < 0.05, Table 7).

**Table 7:** Comparison of pregnancy outcomes

Groups	Pregnancy rate	Pregnancy outcomes			Pregnancy mode	
		Abortion	Premature birth	Full-term birth	Spontaneous pregnancy	In vitro fertilization-embryo transfer
Group A (n = 35)	12 (37.14%)	1 (8.33%)	1 (8.33%)	10 (76.92%)	1 (9.09%)	10 (90.91%)
Group B (n = 35)	16 (44.00%)	1 (6.25%)	1 (6.25%)	14 (87.50%)	2 (13.33%)	13 (86.67%)
Group C (n = 35)	15 (45.71%)	1 (6.67%)	1 (6.67%)	13 (86.67%)	2 (14.29%)	12 (85.71%)
Group D (n = 35)	26 (74.29%)*#$	2 (7.69%)	2 (7.69%)	22 (84.62%)	12 (54.55%)*#$	10 (45.45%)*#$

Note: * *P* < 0.05 vs. group A; # *P* < 0.05 vs. group B; $ *P* < 0.05 vs. group C

## Discussion

Adenomyosis is a poorly understood entity and there is no uniform treatment protocol [4]. From the perspective of treatment, this trial was planned to discover the efficacy of different treatment methods for patients with adenomyosis, and it was finally concluded that ultrasound-guided percutaneous RFA combined with Leuprorelin Acetate had a definite effect on adenomyosis, which could effectively relieve clinical symptoms, effectively protect the postoperative ovarian function, reduce recurrence rate, alleviate pain, and improve the quality of life.

Laparoscopic surgery has emerged as an alternative to laparotomy for focal adenomyosis but it comes with the risk of uterine rupture [18]. According to the detection results in this trial, laparoscopic surgery alone was less effective in the treatment of adenomyosis, which may be related to the unclear boundary between adenomyosis and myometrium to guarantee the complete removal of adenomyosis. Also, it was noted that combined GnRH-a (Leuprorelin Acetate) could improve the efficacy and pregnancy outcomes in adenomyosis patients following laparoscopic surgery. Interestingly, emerging studies have focused on the therapeutic potential of GnRH-a in adenomyosis. For instance, GnRH-a administration in adenomyosis alleviates hypermenorrhea and dysmenorrhea and reduces uterine volume and recurrence possibility [19]. More significantly, it has been also testified that GnRH-a is an effective method in reducing uterine volume in patients with adenomyosis [12]. In fact, the efficacy of combined GnRH-a treatment has been reported in the course of severe adenomyosis, which is manifested as a relief in dysmenorrhea and hypermenorrhea, reduction of uterine volume, and restoration of serum CA125 level [20]. Further, GnRH-a treatment combined with high-intensity focused ultrasound ablation has been found to reduce serum CA125, attenuate adenomyosis lesions, and reduce menstrual blood volume and dysmenorrhea [21]. All these reports support that GnRH-a improves the therapeutic outcome in patients with adenomyosis, which is consistent with our study findings.

As for ultrasound-guided percutaneous RFA, this treatment method showed a greater effect on adenomyosis than laparoscopic surgery. Generally speaking, RFA is a promising minimally invasive organ preservation treatment for adenomyosis [22]. It has been reported that transvaginal ultrasound-guided RFA can reduce uterine volume and symptom severity score in patients with adenomyosis [23]. Concerning a systematic review and meta-analysis, RFA can reduce VAS scores and uterine volume, alleviate dysmenorrhea, and increase pregnancy rates for adenomyosis patients [6]. For patients with adenomyosis who wish to maintain fertility and relieve symptoms, RFA can improve pregnancy outcomes and alleviate pains [24]. Intriguingly, this trial further testified that ultrasound-guided percutaneous RFA combined with GnRH-a (Leuprorelin Acetate) achieved the greatest effect on adenomyosis. In fact, there is evidence confirming that ultrasound-guided transvaginal RFA combined with levonorgestrel-releasing intrauterine system can alleviate dysmenorrhea and symptom severity scores in adenomyosis [25], supporting the outperformance of combined therapy compared with monotherapy. GnRH analogs have been accepted to manage menstrual pain and hypermenorrhea in women with adenomyosis, but drug medications temporarily suppress the menstrual cycle [26]. Considering this, this trial considered a combined therapy to integrate ultrasound-guided percutaneous RFA and GnRH-a analogs to improve the efficacy and pregnancy outcomes in adenomyosis. As expected, ultrasound-guided percutaneous RFA combined with GnRH-a reduced menstrual volume, VAS score, and uterine volume, achieved a total effective rate of 100%, lowered hormone levels and CA125 level, reduced the rate of recurrence, and improved pregnancy outcomes in adenomyosis patients.

In brief, ultrasound-guided percutaneous RFA combined with Leuprorelin Acetate in the treatment of adenomyosis can achieve a better therapeutic effect. Despite this, the trial was conducted with a small sample size, so further validation is needed when the sample size is expanded. From the overall view, this trial supports the efficacy of ultrasound-guided percutaneous RFA combined with GnRH-a to treat adenomyosis, providing a treatment possibility and chance for adenomyosis patients, especially those with reproductive needs.
